# American Surgical Association

**Published:** 1883-07-20

**Authors:** 


### EDITORIALS AND MISCELLANEOUS.

### *AMERICAN SURGICAL ASSOCIATION.*

This Association took a high stand upon the Code of Ethics at Cleveland, notifying their New York members that their resignation would be expected if they rejected the National Code. There are now one hundred members, which is the full number that can be received, under their constitution.

